# Physical exercise during exposure to 40-Hz light flicker improves cognitive functions in the 3xTg mouse model of Alzheimer’s disease

**DOI:** 10.1186/s13195-020-00631-4

**Published:** 2020-05-20

**Authors:** Sang-Seo Park, Hye-Sang Park, Chang-Ju Kim, Hyun-Sik Kang, Dong-Hyun Kim, Seung-Soo Baek, Tae-Woon Kim

**Affiliations:** 1 Department of Physiology, College of Medicine, KyungHee University, Seoul, Republic of Korea; 2 Department of Kinesiology, College of Public Health and Cardiovascular Research Center, Lewis Katz school of Medicine, Temple University, Philadelphia, PA USA; 3 College of Sports science, Sungkyunkwan University, Suwon, Republic of Korea; 4 Department of Exercise & Health Science, Exercise Rehabilitation Research Institute, Sangmyung University, Seoul, Republic of Korea

**Keywords:** Alzheimer’s disease, Exercise, Amyloid beta, Tau, 40-Hz light flicker, Cognitive function, Mitochondria, Apoptosis, Neuroplasticity, Hippocampus

## Abstract

**Background:**

Exercise promotes brain health and improves cognitive functioning in the elderly, while 40-Hz light flickering through the visual cortex reduces amyloid beta (Aβ) by stabilizing gamma oscillation. We examined whether exercise was associated with hippocampus-mediated improvement in cognitive functioning in the 3xTg-Alzheimer’s disease (3xTg-AD) murine model following exposure to 40-Hz light flickering and exercise.

**Methods:**

We subjected 12-month-old 3xTg-AD mice to exercise and 40-Hz light flickering for 3 months to investigate spatial learning, memory, long-term memory, Aβ levels, tau levels, mitochondrial functioning including Ca^2+^ retention and H_2_O_2_ emission, apoptosis, and neurogenesis in the hippocampus.

**Results:**

Treatments had a positive effect; however, the combination of exercise and 40-Hz light flickering exposure was most effective in reducing Aβ and tau levels. Reducing Aβ and tau levels by combination of exercise and 40-Hz light flickering improves Ca^2+^ homeostasis and reactive oxygen species such as H_2_O_2_ in mitochondria and apoptosis including bax, bcl-2, cytochrome c, and cleaved caspase-3 and cell death, cell differentiation, and neurogenesis in the 3xTg-AD model of the hippocampus, resulting in improving cognitive impairment such as spatial learning, memory and long term memory.

**Conclusion:**

Our results show that exercising in a 40-Hz light flickering environment may improve cognitive functioning by reducing Aβ and tau levels, thereby enhancing mitochondrial function and neuroplasticity.

## Background

Alzheimer’s disease (AD) is the most common degenerative brain disease. AD causes dementia, whose progressive onset leads to a gradual worsening of cognitive functioning, including memory. In the initial stages of AD, problems with memory related to recent events occur with alterations in cognitive function such as language ability and judgment, which eventually lead to the complete loss of motor function. AD accounts for > 70% of all dementia cases [1]. AD is associated with various cellular changes in the brain, including synaptic alterations, mitochondrial structural and functional changes, abnormal inflammatory responses, extracellular amyloid beta (Aβ) accumulation, and intracellular neurofibrillary tangles [2–4]. In particular, it is known that Aβ is directly correlated with AD pathology and that longer isoforms with 42–43 residues (the smaller isoform has 40 residues) cause neurodegeneration and cognitive dysfunction, eventually progressing to dementia [5, 6]. Reduced Aβ clearance or Aβ overproduction may cause Aβ accumulation in subcellular compartments, including synapses and mitochondria, and may impair organelle and ultimately neuronal function [7–9].

AD is caused by hippocampal atrophy, which is involved in memory and learning, the presence of senile plaques, and the accumulation of hyperphosphorylated aggregates of tau protein [10, 11]. Overexpression or hyperphosphorylation of tau protein due to AD impairs axonal migration of organelles including the mitochondria [12, 13]. There is substantial evidence suggesting that mitochondrial dysfunction is associated with aging and neurodegenerative diseases. Reduced mitochondrial function has been demonstrated using AD transgenic mouse models as well as post-mortem brain tissue of patients with AD [14, 15], fibroblasts, and blood cells [16–18]. Mitochondrial dysfunction participates in the progression of AD and is present in all stages of the disease. It has been suggested that AD is not limited to the brain but could be a systemic disease [16].

In the brain, gamma oscillation occurs between 25 and 145 Hz, affecting various behavioral functions such as attention and memory [19]. This is disrupted in animal models and clinical studies of AD and other disorders [20–22]. Specifically, in the brains of AD patients, gamma rhythms are known to be interrupted and Aβ has been suggested as the potential cause, particularly in the hippocampus [23]. Aβ accumulation affects memory through the inhibition of electrical signaling such as gamma oscillations, which play an important role in cognitive functioning and sensory response [24–26]. The wavelength, duration, and intensity of light exposure regulate the cognitive tasks that the brain responds to, and these light responses have been observed in subcortical areas, such as the hypothalamus, the brain stem, and the thalamus, as well as in limbic areas including the amygdala and the hippocampus [27]. According to Naeser et al. [28], cognitive function is improved through light-emitting diode (LED) therapy in patients with chronic traumatic brain injury. In addition, previous studies have consistently reported that exercise is beneficial to brain functioning and is a primary method of preventing and treating AD in combination with drugs. However, unlike certain drugs that target localized causes, the positive effects of exercise mitigate or delay multiple aspects related to AD. In animal studies using familial AD gene mutations, exercise was reported to minimize neurotoxicity caused by AD neuropathy and stimulate neuronal regeneration, contributing to an improvement in cognitive functioning through a reduction in beta-secretase activity [29], decreased accumulation of amyloid plaques and soluble Aβ [30, 31], and decreased pTau [32]. Therefore, this study aimed to examine the effects of exercise training in a 40-Hz light flicker environment on Aβ accumulation in the hippocampus, Akt/tau, mitochondrial function, neuroplasticity, and cognitive functioning in an AD animal model.

## Methods

### Animals

All animal experiments were performed in accordance with the guidelines of the National Institutes of Health and the Korean Academy of Medical Science. The study protocol was approved by the KyungHee University Institutional Animal Care and Use Committee (approval number KHUASP [SE]-17-103). The mice were housed under conditions of controlled temperature (25 ± 1 °C) and lighting (7 am to 7 pm) with food and water ad libitum. Fifteen-month-old male wild-type and 3xTg mice were randomly divided into a wild-type control group (CON), a 3xTg-AD group (AD), a 3xTg-AD and 40-Hz light flickering group (AD+40), a 3xTg-AD and Exercise group (AD+EX), and a 3xTg and exercise under 40-Hz light flickering group (AD+40+EX) (*n* = 10 in each group). We used the 3xTg-AD mice harboring APP Swe, PS1M146V, and tau P301L human transgenes. The genotype was confirmed by PCR analysis of DNA obtained by tail biopsies. BrdU (Sigma, St. Louis MO, USA) was administered intraperitoneally (i.p.) at 100 mg/kg/day for 7 days, and we sacrificed the mice 4 weeks after first day of BrdU injection to observe neurogenesis.

### Exercise protocol and 40-Hz light flickering of exposure time

Exercise sessions began at age 12 months of 3xTg mice. The exercise groups exercised on a treadmill made for animal use once daily in the dark, 6 days per week for 12 consecutive weeks. For exercises of adaption, mice were allowed 5 min of warm up at a 0° inclination at 3 m/min, 30 min of the main exercise at 10 m/min, and 5 min of cool down at 3 m/min which were performed for the first 3 weeks. Subsequently, mice were subjected to 40 min of the main exercise at 11 m/min for weeks 4 to 6, 50 min of the main exercise at 12 m/min for weeks 7 to 9, and 50 min of the main exercise at 13 m/min for the final weeks 10 to 12. Exposure time of 40-Hz light flickering was the same as exercise time.

### Behavioral tests

#### Morris water maze

The Morris water maze task was used to assess spatial learning and working memory. One day before training, the mice were habituated to swimming for 60 s in the pool without a platform. All mice were trained three times per day for five consecutive days. A probe trial was performed 24 h after the final training session. When a mouse found the platform, it was allowed to remain there for 30 s. If the mouse did not find the platform within 60 s, it was guided by hand to the platform. The mice underwent a 60-s retention probe test, after which the platform was removed from the pool. The data were automatically collected using the Smart Video Tracking System (Smart version 2.5, Panlab, Barcelona, Spain).

#### Step through avoidance test

The step through avoidance task was used to assess long-term memory. For the training period, the mouse was placed at the entrance identified by the halogen bulbs and the door of the box was opened. When the mouse entered the dark place, the door and the mouse were allowed to stay for 20 s. This training was repeated twice. Finally, during the third training session, when the mouse entered the dark place, the door closed at the same time and the mouse received a 1-mA scramble foot shock for 2 s. After 24 h of the foot shock, mice were placed back at the entrance identified by the halogen bulb, and when the door opened, the time taken for mice to enter the dark place was measured. A latency time > 300 s was counted as 300 s.

#### Preparation of tissue

The mice were euthanized immediately after the behavior test. To prepare the brain slices, the animals were fully anesthetized with ethyl ether, perfused transcardially with 50 mM phosphate-buffered saline (PBS), and then fixed with a freshly prepared solution of 4% paraformaldehyde in 100 mM phosphate buffer (pH 7.4). The brains were then removed, post-fixed in the same fixative overnight, and transferred into a 30% sucrose solution for cryoprotection. Coronal sections with a thickness of 40 μm were created using a freezing microtome (Leica, Nussloch, Germany). From each group of 10 animals, 5 were used for immunohistochemistry and 5 for western blot and mitochondrial function analysis. The hippocampal tissue for western blot analysis was immediately stored at − 70 °C until use. For immunohistochemistry, 2 sections from each group were analyzed, resulting in a total of 10 slices.

#### Immunohistochemistry

To visualize cell differentiation, immunohistochemistry was performed for DCX staining in the dentate gyrus (DG) and Aβ in CA1 to 3 and in the DG hippocampus. The sections were incubated in PBS for 10 min and then washed three times for 3 min in the PBS. The sections were then incubated in 1% H_2_O_2_ for 15 to 30 min. The sections were selected from each brain and incubated overnight with goat anti-DCX antibody (1:500; Santa Cruz) and mouse purified anti-β-amyloid antibody (1:200; Biolegend) and then with biotinylated rabbit secondary antibody (1:250; Vector Laboratories) for another 90 min. The secondary antibody was amplified with the Vector Elite ABC Kit® (1100; Vector Laboratories). Antibody-biotin-avidin-peroxidase complexes were visualized using the 3,3,diaminobenzidine (DAB) substrate kit (Vector Laboratories). The slides were air-dried overnight at room temperature, and the coverslips were mounted using Permount®.

#### Immunofluorescence

BrdU/NeuN-positive cells in the DG were tested for immunofluorescence. In brief, the brain sections were permeabilized by incubation in 0.5% Triton X-100 in PBS for 20 min, incubated in 50% formamide-2× standard saline citrate at 65 °C for 2 h, denatured in 2 N HCl at 37 °C for 30 min, and then rinsed twice in 100 mM sodium borate (pH 8.5), in that order. The sections were incubated overnight with rat anti-BrdU antibody (1:200; Abcam, Cambridge, UK) and mouse anti-NeuN antibody (1:200; Millipore, Temecula, CA). The brain sections were then washed in PBS and incubated with the appropriate secondary antibodies for 1 h. The secondary antibodies used were anti-mouse IgG Alexa Fluor-488 and anti-rat IgG Alexa Fluor-560. Images were captured using an FV3000 confocal microscope (Olympus, Tokyo, Japan).

#### TUNEL staining

To visualize DNA fragmentation, we performed TUNEL staining using an In Situ Cell Death Detection Kit (Roche Diagnostics, Risch-Rotkreuz, Switzerland) according to the manufacturer’s protocol. Sections were post-fixed in ethanol-acetic acid (2:1), rinsed, incubated with proteinase K (100 mg/mL), and then rinsed again. Next, they were incubated in 3% H_2_O_2_, permeabilized with 0.5% Triton X-100, rinsed again, and incubated in the TUNEL reaction mixture. The sections were rinsed and visualized using Converter-POD with 0.03% DAB, counterstained with Cresyl violet, and mounted onto gelatin-coated slides. The slides were air-dried overnight at room temperature and cover-slipped using Permount mounting medium.

#### Western blotting

Hippocampal tissues were homogenized on ice and lysed in lysis buffer containing 50 mM Tris–HCl (pH 7.5), 150 mM NaCl, 0.5% deoxycholic acid, 1% Nonidet P40, 0.1% sodium dodecyl sulfate, 1 mM PMSF, and leupeptin 100 mg/mL. The protein content was measured using a colorimetric protein assay kit (Bio-Rad, Hercules, CA). Thirty micrograms of protein was separated on sodium dodecyl sulfate-polyacrylamide gels and transferred onto a nitrocellulose membrane, which was incubated with mouse β-actin (1:1000; Santa Cruz Biotechnology), GAPDH (1:3000; Santa Cruz Biotechnology), t-Akt and p-Akt (1:1000; Cell Signaling), t-GSK3β and p-GSK3β (ser 9) (1:1000; Cell Signaling), t-Tau and p-Tau (ser202/Thr205 (1:1000; Thermo Fisher), Bcl-2 and cytochrome C (1:1000; Santa Cruz Biotechnology), Bax (1:1000; Cell Signaling), cleaved caspase-3 (1:700; Cell Signaling), brain-derived neurotrophic factor (BDNF; 1:1000; Alomone), PSD95 (1:1000; Cell Signaling), and synaptophysin (1:1000; Abcam) primary antibodies. Horseradish peroxidase-conjugated secondary anti-mouse antibodies were used for Bcl-2, p-Tau, cytochrome C, β-actin, and GAPDH; anti-rabbit conjugated secondary antibodies were used for t-Akt, p-Akt, t-tau, p-tau, t-GSK3β, Bax, cleaved caspase-3, BDNF, PSD95, and synaptophysin.

#### Mitochondrial Ca^2+^ retention capacity

The mitochondrial calcium retention capacity was tested to assess the susceptibility of the permeability transition pore (PTP) to opening. Briefly, after grinding the hippocampal tissue, overlaid traces of changes in fluorescence induced by Calcium Green-5 N were measured continuously (ΔF/min) at 37 °C during state 4 respiration using a Spex FluoroMax 4 spectrofluorometer (Horiba Scientific, Edison, NJ). After establishing the background ΔF (hippocampal tissue in the presence of 1 μM is Calcium Green-5N, 1 U/mL hexokinase, 0.04 mM EGTA, 1.5 nM thapsigargin, 5 mM 2-deoxyglucose, 5 mM glutamate, 5 mM succinate, and 2 mM malate), the reaction was initiated by addition of Ca^2+^ pulses (12.5 nM), with excitation and emission wavelengths set at 506 nm and 532 nm, respectively. The total mitochondrial Ca^2+^ retention capacity prior to PTP opening (i.e., release of Ca^2+^) was expressed as pmol/mg tissue weight.

#### Mitochondrial H_2_O_2_ emission

H_2_O_2_ emission was measured at 37 °C (Δ*F*/min) during state 4 respiration (10 μg/ml oligomycin) by continuously monitoring oxidation of Amplex Red (excitation/emission *λ* = 563/587 nm) using a Spex FluoroMax 4 spectrofluorometer with the following protocol: 10 μM Amplex Red, 1 U/mL horseradish peroxidase, and 10 μg/mL oligomycin settings and 1 mM malate + 2 mM glutamate (complex I substrates), 3 mM succinate (complex II substrate), and 10 mM glycerol-3-phosphate (lipid substrate). The H_2_O_2_ emission rate after removing the background value from each of the standard values (standard curve) was calculated from the Δ*F*/min gradient values and expressed as pmol/min/mg tissue weight.

### Statistical analysis

Spatial learning was measured by two-way mixed analysis of variance to account for repeated measures (5 groups; between × 5 days repeated; within). Cell counting and optical density quantification for Aβ, TUNEL, DCX, and BrdU/NeuN-positive cells’ expression were performed using Image-Pro® Plus (Media Cyberbetics Inc.) attached to a light microscope (Olympus, Tokyo, Japan). The data were analyzed with one-way ANOVA, followed by the Duncan post hoc tests. All values are expressed as the mean ± standard error of the mean (S.E.M.), and *P* values < .05 were considered significant.

## Results

### Effect of exercise under exposure to the 40-Hz light flickering on spatial working learning and memory and long-term memory

**Fig. 1:**
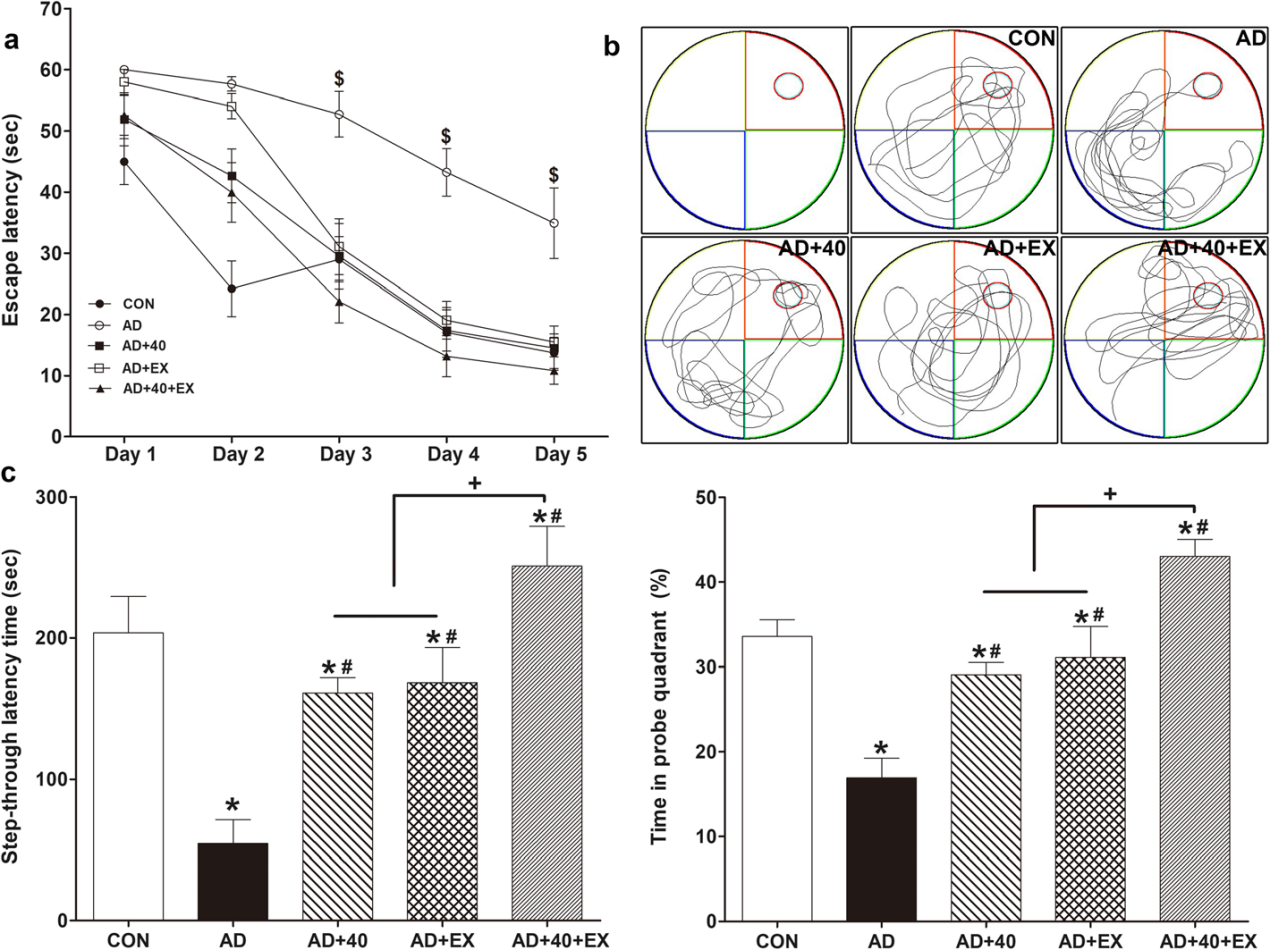
Effects of exercise under exposure to the 40-Hz light flicker on spatial learning and memory and long-term memory. The Morris water maze task for spatial learning (**a**) and memory (**b**) and step through task for long-term memory (**c**). CON: wild-type, AD: 3xTg-AD, AD+40: 3xTg-AD and 40-Hz light flickering, AD+EX: 3xTg-AD and exercise, and AD+40+EX: 3xTg-AD and exercise in the 40-Hz light flickering group. Data are expressed as the mean ± standard error of the mean (SEM). *P < .05 compared to the CON group. ^#^*P < .*05 compared to the AD group. ^+^*P < .*05 compared to the AD+40+EX. ^$^*P* < .05 for a difference between the group because of the interaction effect

The Morris water maze and step through avoidance task were performed to assess spatial learning and memory and long-term memory. Spatial learning was assessed as the time spent on the platform. In the two-way mixed ANOVA accounting for the repeated measures of spatial learning, results are presented as the outcome of a multivariate test. During the Morris water maze task, there was a significant interaction between the repeated measure and group (day; *p* = .000, day × group; *p* = .000). Over time, the effect of repeated learning had differential effects on the groups. Particularly, significant decreases in the time taken to find the platform in spatial learning capability during the Morris water maze task were observed from days 3 to 5 of training in the AD+EX, AD+40, and AD+40+EX groups: day 1, CON vs AD (*P = .*027); day 2, CON vs AD (*P = .*000), CON vs AD+40 (*P = .*019), CON vs AD+EX (*P <* .001), and AD vs AD+40+EX (*P = .*019); day 3, CON vs AD (*P = .*003), AD vs AD+40 (*P = .*004), AD vs AD+EX (*P = .*015), and AD vs AD+40+EX (*P <* .001); day 4, CON vs AD (*P <* .001), AD+40 (*P <* .001), AD vs AD+EX (*P = .*001), and AD vs AD+40+EX (*P = .*000); and day5, CON vs AD (*P <* .001), AD vs AD+40 (*P <* .001), AD vs AD+EX (*P = .*003), and AD vs AD+40+EX (*P <* .001). In the AD group, the implementation of exercise and 40-Hz light flickering was effective in the repetitive learning, especially the combination of 40-Hz light flickering and exercise. Spatial memory (*P <* .001) and long-term memory (*P <* .001) were significantly reduced in the AD group compared with the CON group. In contrast, spatial memory and long-term memory were enhanced by exposure to AD+40-Hz (*P =* .008, *P =* .030), AD+EX (*P =* .001, *P =* .019), and AD+40+EX (*P <* .001 respectively). Groups of treatment comparison revealed significant differences for spatial learning and long-term memory between AD+40+EX and AD+40 (*p* < .001, *p* = .001, respectively) and AD+EX (*P = .*007, *P = .*002, respectively) groups. AD+40 and AD+EX groups did not show a significant intergroup difference. Therefore, it is worth noting that exercise was most effective in improving cognitive functioning under the 40-Hz light flickering condition in AD and that the results were better than the normal aging group used as CON (Fig. 1, Table 1).

**Table 1:** Effect of exercise under exposure to the 40-Hz light flickering on spatial learning and memory and long-term memory

	Spatial learning training (s)					SM (%)	LTM (s)
Group	1 day	2 days	3 days	4 days	5 days		
CON	45.00 ± 3.76	24.20 ± 4.54	29.00 ± 3.71	17.07 ± 4.06	13.73 ± 2.54	33.57 ± 1.97	203.60 ± 23.73
AD	60.00 ± 0.0*	57.67 ± 1.17*	52.75 ± 3.71*	43.25 ± 3.88*	34.92 ± 5.78*	16.93 ± 2.31*	54.40 ± 16.90*
AD+40	51.93 ± 4.33	42.67 ± 4.40*	29.53 ± 5.34^#^	17.40 ± 3.37^#^	14.53 ± 3.63^#^	29.10 ± 1.42*^#^	161.20 ± 10.95*^#^
AD+EX	58.00 ± 2.05	54.05 ± 2.05*	31.08 ± 4.58^#^	19.08 ± 3.06^#^	15.50 ± 1.32^#^	31.08 ± 3.66*^#^	168.40 ± 24.86*^#^
AD+40+EX	52.56 ± 3.22	39.94 ± 4.87*	22.11 ± 3.48^#^	13.17 ± 3.38^#^	10.83 ± 2.22^#^	43.01 ± 2.05*^#+^	250.83 ± 28.29*^#+^

*P < .05 compared to the CON group, ^#^*P* < .05 compared to the AD group, ^+^*P* < .05 compared to the AD+40 and AD+EX. *SM* spatial memory, *LTM* long-term memory

### Effects of exercise under exposure to 40-Hz light flickering on Aβ in the hippocampus

**Fig. 2:**
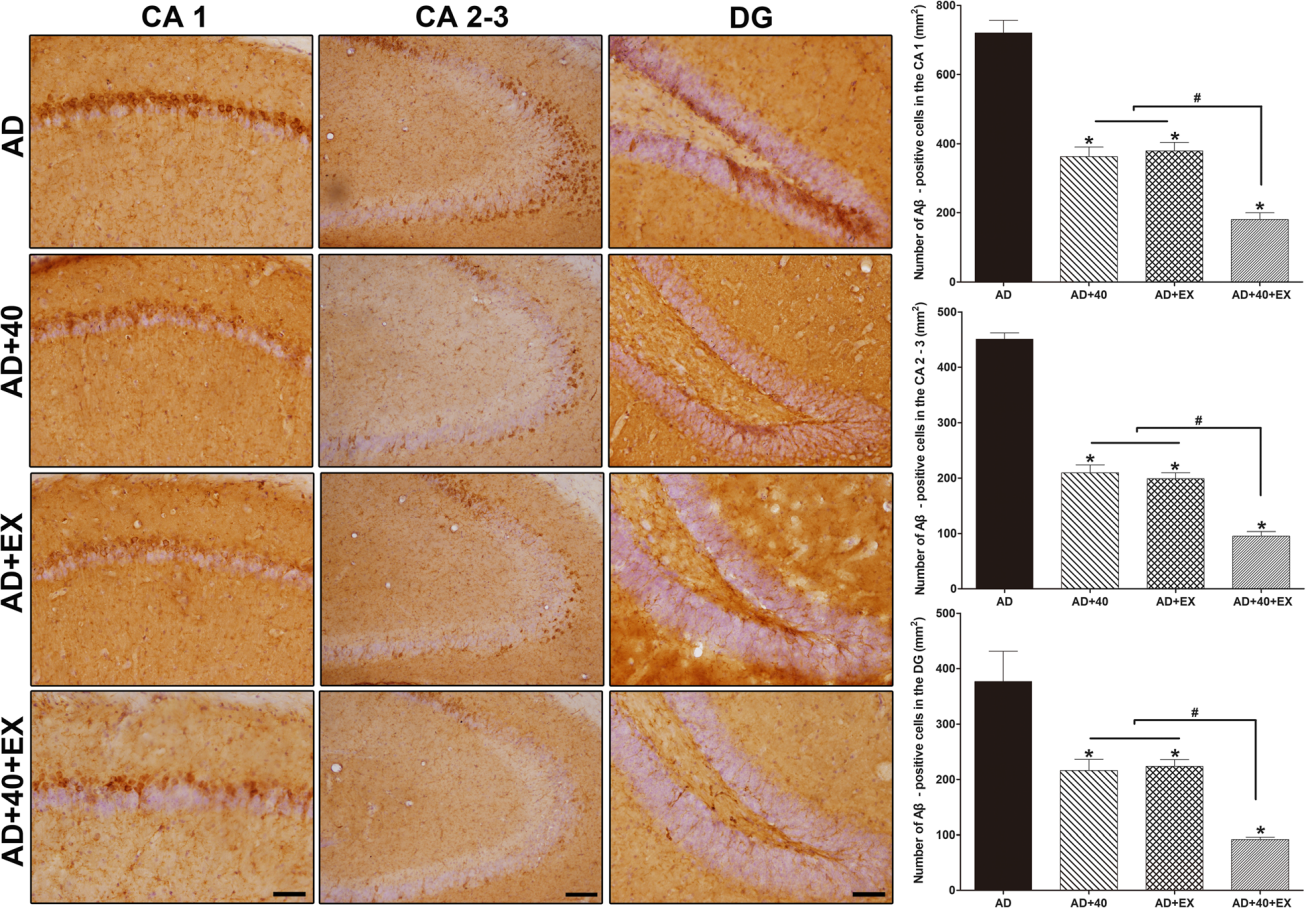
Effects of exercise under exposure to the 40-Hz light flicker on Aβ in the hippocampus. Photomicrographs and data of Aβ-positive cells. The scale bar represents 50 μm. AD: 3xTg-AD, AD+40: 3xTg-AD and 40-Hz light flickering, AD+EX: 3xTg-AD and exercise, and AD+40+EX: 3xTg-AD and exercise in the 40-Hz light flickering. Data are expressed as the mean ± standard error of the mean (SEM). *P < .05 compared to the AD group. ^+^*P < .*05 compared to the AD+40+EX

The number of Aβ-positive cells in the CA1, CA2–3, and DG of the hippocampus was significantly decreased in the AD group compared with the treatment groups; Aβ-positive cells were reduced in the AD+40 (CA1: *P <* .001, CA2–3: *P <* .001, DG: *P <* .001), AD+EX (CA1: *P <* .001, CA2–3: *P <* .001, DG: *P <* .001), and AD+40+EX (CA1: *P <* .001, CA2–3: *P <* .001, DG: *P* < .001) groups. When exercise alone was compared with exercise performed under the 40-Hz light flickering condition, the AD+40+EX group showed an intergroup difference when compared to AD+40 (*P <* .001) and AD+EX (*P <* .001) across all areas of the hippocampus (CA1, CA2–3, and DG); however, groups exposed to 40-Hz light flickering or exercise alone (AD+40 and AD+EX groups, respectively) did not show any significant intergroup difference. Therefore, each method was effective in removing Aβ, although performing exercise under the 40-Hz light flickering condition was the most efficacious. It is interesting to note that exercise during the 40-Hz light flickering condition reduced Aβ to CON levels in the CA2–3 and DG of the hippocampus (Fig. 2, Table 2).

**Table 2:** Effect of exercise under exposure to the 40-Hz light flickering on Aβ in the hippocampus

Group	CA1 (mm^2^)	CA2–3 (mm^2^)	DG (mm^2^)
AD	720.32 ± 36.70	450.88 ± 11.23	376.24 ± 29.76
AD+40	362.66 ± 28.02*	209.51 ± 14.34*	216.23 ± 20.14*
AD+EX	379.40 ± 29.47*	191.92 ± 10.67*	223.64 ± 12.01*
AD+40+EX	180.35 ± 7.68*^#^	95.32 ± 7.87*^#^	91.87 ± 4.48*^#^

*P < .05 compared to the AD group, ^#^*P* < .05 compared to the AD+EX and AD+ 40 group

### Effects of exercise under exposure to the 40-Hz light flickering on Akt/tau in the hippocampus

**Fig. 3:**
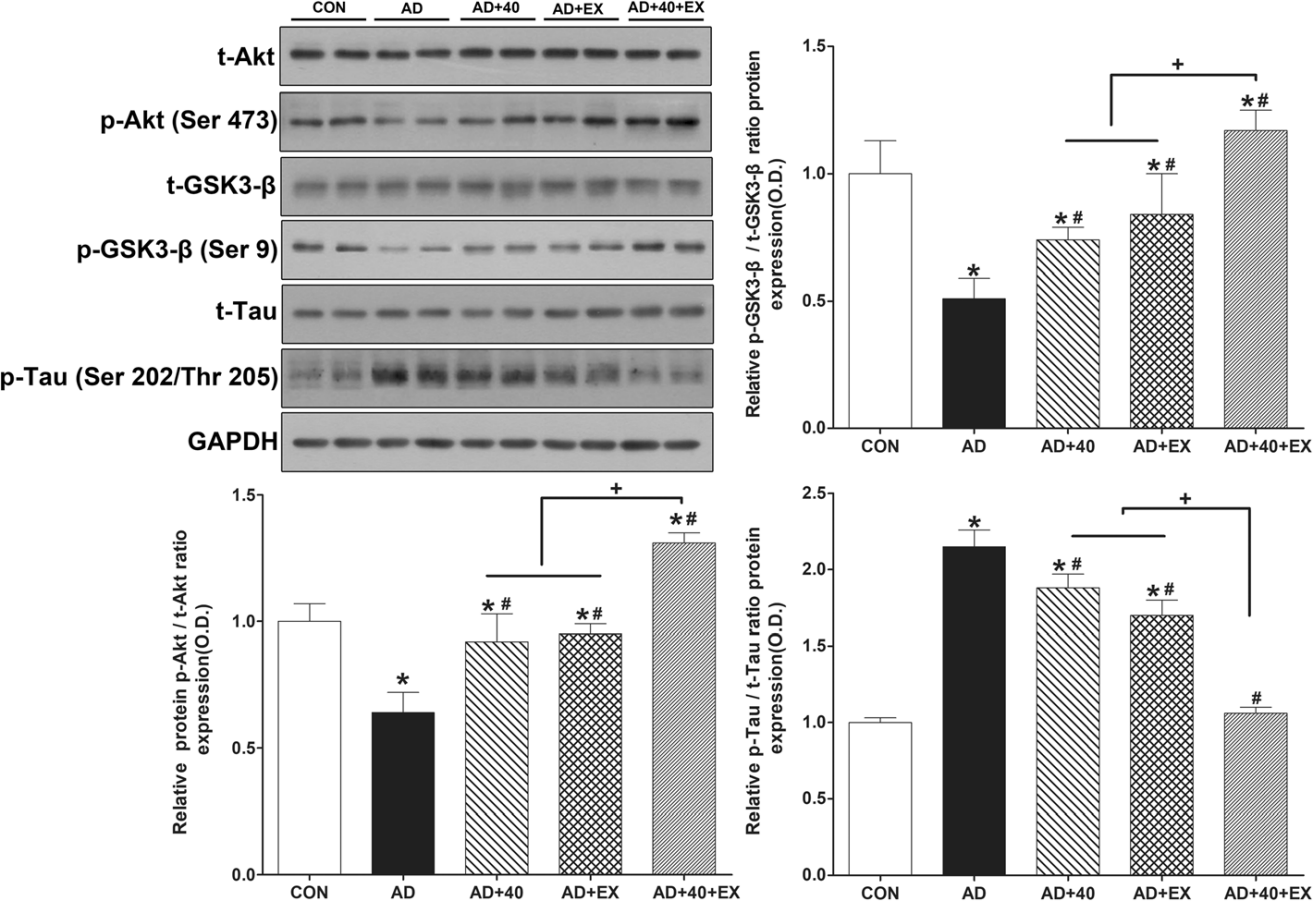
Effects of exercise under exposure to the 40-Hz light flicker on p-Akt/t-Akt, p-GSK3β/t-GSK3β, and p-tau/t-tau ratio in the hippocampus. CON: wild-type, AD: 3xTg-AD, AD+40: 3xTg-AD and 40-Hz light flickering, AD+EX: 3xTg-AD and exercise, and AD+40+EX: 3xTg-AD and exercise in the 40-Hz light flickering. Data are expressed as the mean ± standard error of the mean (SEM). *P < .05 compared to the CON group. ^#^*P < .*05 compared to the AD group. ^+^*P < .*05 compared to the AD+40+EX

Western blot was used to analyze the changes in expression of Akt/tau proteins in the hippocampus. For an intergroup comparison, the ratio of the CON group was set to 1 and a comparison was made with the relative value of each group. When the CON group was compared to the AD group, the p-Akt/Akt ratio (*P <* .001) and p-GSK3β/GSK3β ratio (*P <* .001) were reduced while the p-tau/tau ratio (*P <* .001) increased. In contrast, when the AD group was compared to the treatment groups, the p-Akt/Akt ratio and p-GSK3β/GSK3β ratio of protein expression were increased: AD+40 (p-Akt/Akt ratio: *P <* .001, p-GSK3β/GSK3β ratio: *P =* .003), AD+EX (p-Akt/Akt ratio: *P* < .001, p-GSK3β/GSK3β ratio: *P =* .001), and AD+40+EX (p-Akt/Akt ratio: *P < .*001, p-GSK3β/GSK3β ratio: *P <* .001). The p-Tau/tau ratio expression was significantly reduced in AD+40 (*P =* .026), AD+EX (*P =* .001), and AD+40+EX (*P <* .001). When a comparison was made among treatment groups, AD+40+EX showed an intergroup difference with AD+40 (*P =* .026) and AD+EX (*p* < 0.001), while there was no difference between AD+40 and AD+EX groups. In other words, 40-Hz light flickering and exercise were both effective in reducing the Akt/tau protein ratio when administered alone, but exercise under the exposure to 40-Hz light flickering was even more effective, reducing the level to that of the CON group. The p-Akt/Akt and p-GSK3β/GSK3β ratios were also significantly increased compared to the CON group (Fig. 3, Table 3).

**Table 3:** Effect of exercise under exposure to the 40-Hz light flickering on Akt/Tau in the hippocampus

Group	p-Akt/Akt ratio	P-GSK3β/GSK3β ratio	p-Tau/tau ratio
CON	1.00 ± 0.07	1.00 ± 0.13	1.00 ± 0.03
AD	0.57 ± 0.05*	0.48 ± 0.05*	2.15 ± 0.11*
AD+40	0.80 ± 0.15*^#^	0.75 ± 0.04*^#^	1.88 ± 0.09*^#^
AD+EX	0.84 ± 0.04*^#^	0.77 ± 0.05*^#^	1.70 ± 0.10*^#^
AD+40+EX	1.24 ± 0.04*^#+^	1.16 ± 0.07*^#+^	1.06 ± 0.04*^#+^

^*^P < .05 compared to the CON group, ^#^*P* < .05 compared to the AD group, ^+^*P* < .05 compared to the AD+40 and AD+EX

### Effects of exercise under exposure to the 40-Hz light flickering on Ca^2+^ retention and H_2_O_2_ production in the hippocampus

**Fig. 4:**
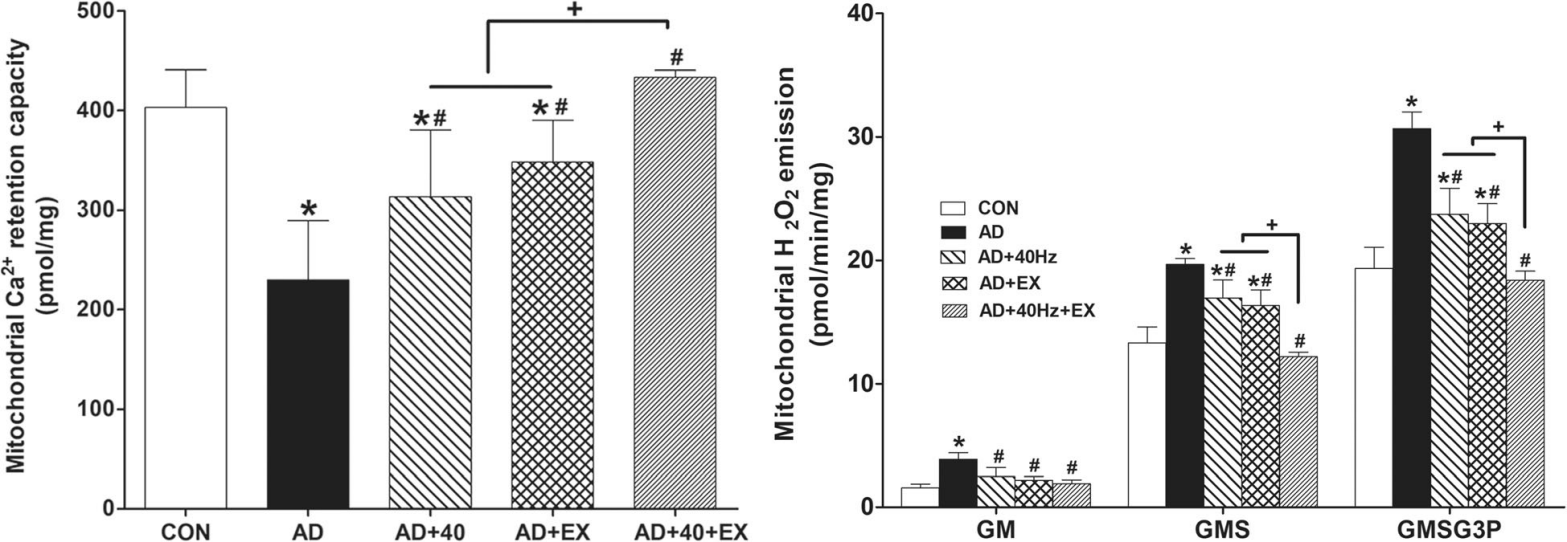
Effects of exercise under exposure to the 40-Hz light flicker on mitochondrial functions in the hippocampus. Control (CON): wild-type, AD: 3xTg-AD, AD+40: 3xTg-AD and 40-Hz light flickering, AD+EX: 3xTg-AD and exercise, and AD+40+EX: 3xTg-AD and exercise in the 40-Hz light flickering. Data are expressed as the mean ± standard error of the mean (SEM). *P < .05 compared to the CON group. ^#^*P < .*05 compared to the AD group. ^+^*P < .*05 compared to the AD+40+EX

Mitochondrial Ca^2+^ retention capacity in the hippocampus was reduced in the AD group when compared with the CON group (*P <* .001). In contrast, when the AD group was compared to the treatment groups, AD+40 (*P =* .006), AD+EX (*P <* .001), and AD+40+EX (*P <* .001) showed increased mitochondrial Ca^2+^ retention capacity. An intergroup comparison revealed an intergroup difference between the AD+40 (*P <* .001) and AD+EX (*P <* .001) groups, while the AD+40 and AD+EX groups did not show any significant difference. The mitochondrial H_2_O_2_ production rate was calculated using the complex I substrate (glutamate + malate, GM), the complex 2 substrate (succinate, GMS), and the lipid substrate (glycerol-3 phosphate, GMSG3P). The mitochondrial H_2_O_2_ production rate in the hippocampus was significantly increased compared with the CON group (GM: *P =* .007, GMS: *P <* .001, GMSG3P: *P* < .001). In contrast, when the AD group was compared to the treatment groups, H_2_O_2_ production was significantly reduced in the AD+40 (GM: *P =* .027, GMS: *P <* .001, GMSG3P: *P =* .005), AD+EX (GM: *P =* .034, GMS: *P <* .001, GMSG3P: *P =* .003), and AD+40+EX (GM: *P =* .019, GMS: *P <* .001, GMSG3P: *P <* .001) groups. In the intergroup comparisons, the GM did not show a difference among the treatment groups, but a significant difference was observed for GMS and GMSG3P expression between the AD+40+EX and AD+40 (*P =* .025, *P =* .005, respectively) and AD+EX groups (*P =* .012, *P =* .009, respectively). However, AD+40 and AD+EX groups did not show any significant difference. Thus, exercise under exposure to 40-Hz light flickering appeared to be the most effective in improving mitochondrial function in the hippocampus by maintaining mitochondrial Ca^2+^ retention and reducing ROS. These levels were recovered to normal levels as in the CON group (Fig. 4, Table 4).

**Table 4:** Effect of exercise under exposure to the 40-Hz light flickering on Ca^2+^ retention and H_2_O_2_ in the hippocampus

Group	Ca^2+^retention (pmol/mg)	H_2_O_2_ emission (pmol/min/mg)		
		GM	GMS	GMSG3P
CON	311.42 ± 17.52	3.07 ± 0.30	24.16 ± 1.14	31.82 ± 2.22
AD	158.41 ± 10.60*	4.80 ± 0.24*	39.08 ± 1.57*	48.68 ± 1.09*
AD+40	230.28 ± 6.39*^#^	3.32 ± 0.28^#^	30.65 ± 1.06*^#^	39.68 ± 0.79*^#^
AD+EX	252.60 ± 14.34*^#^	3.37 ± 0.48^#^	31.16 ± 0.70*^#^	39.16 ± 0.70*^#^
AD+40+EX	344.93 ± 16.99*^#+^	3.25 ± 0.38^#^	25.44 ± 1.02*^#+^	30.73 ± 2.43*^#+^

*P < .05 compared to the CON group, ^#^*P* < .05 compared to the AD group, ^+^*P* < .05 compared to the AD+40 and AD+EX

### Effects of exercise under exposure to the 40-Hz light flickering on apoptosis and cell death in the hippocampus

**Fig. 5:**
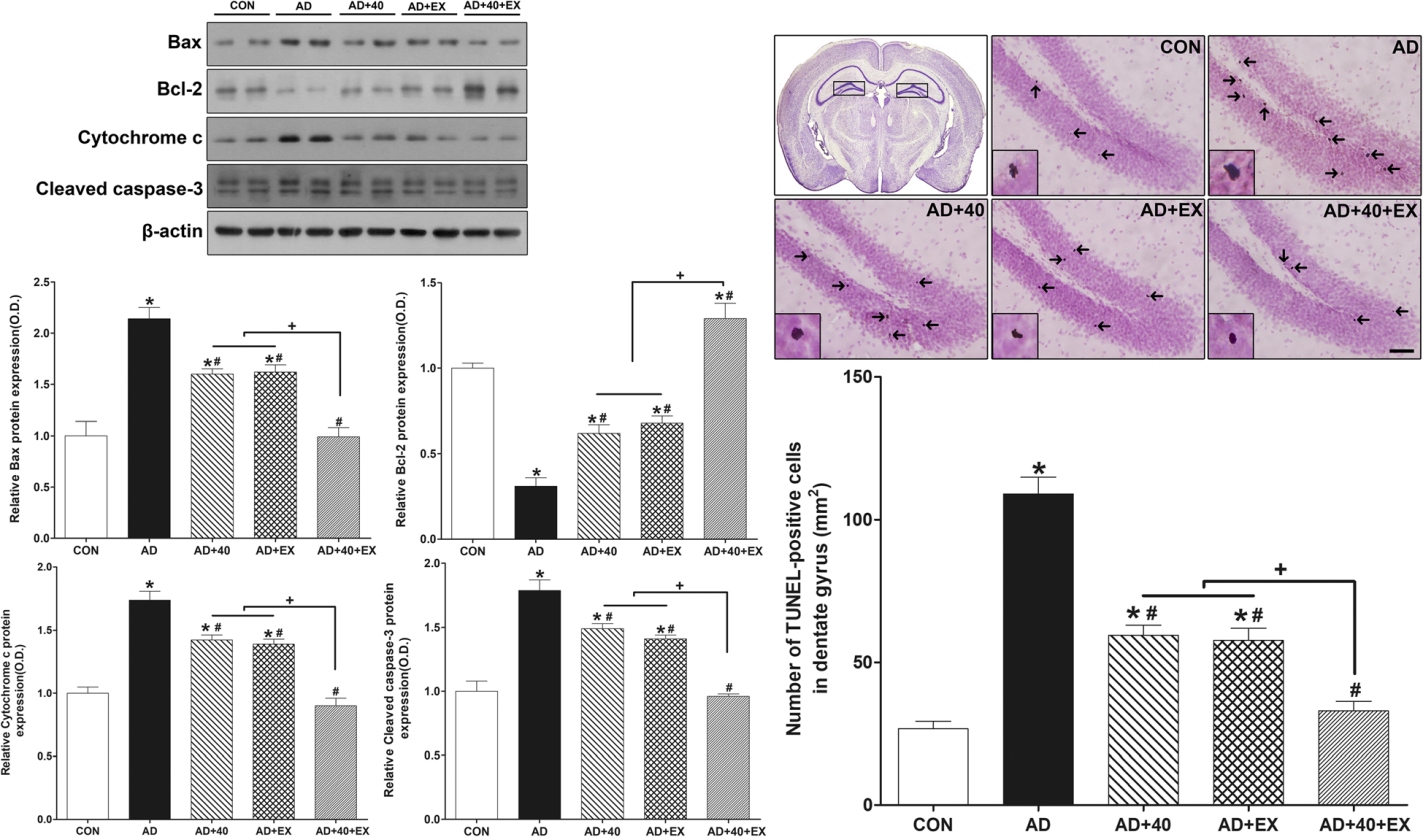
Effects of exercise under exposure to the 40-Hz light flicker on apoptosis and cell death in the hippocampus. Photomicrographs and data of TUNEL-positive cells. The scale bar represents 50 μm. Control (CON): wild-type, AD: 3xTg-AD, AD+40: 3xTg-AD and 40-Hz light flickering, AD+EX: 3xTg-AD and exercise, and AD+40+EX: 3xTg-AD and exercise in the 40-Hz light flickering. Data are expressed as the mean ± standard error of the mean (SEM). *P < .05 compared to the CON group. ^#^*P < .*05 compared to the AD group. ^+^*P < .*05 compared to the AD+40+EX

In order to examine the changes in apoptosis in the hippocampus, we analyzed cell death using TUNEL-positive cells and the expression of Bax, Bcl-2, cytochrome C, and cleaved caspase-3 proteins. For intergroup comparison of apoptosis, the value of the CON group was set to 1 and compared with the relative value of each group. When the CON and AD groups were compared, the expression of Bax (*P <* .001), cytochrome C (*P =* .001), and cleaved caspase-3 (*P <* .001) increased, while Bcl-2 (*P <* .001) was reduced. In contrast, when the AD group was compared with the treatment groups, significant differences were observed with the following treatment groups: AD+40 (Bax: *P =* .001, Bcl-2: *P =* .009, cytochrome C: *P =* .038, cleaved caspase-3*P =* .002), AD+EX (Bax: *P =* .001, Bcl-2: *P =* .002, cytochrome c: *P =* .013, cleaved caspase-3: *P <* .001), and AD+40+EX (*P <* .001 respectively). In the comparison among the treatment groups, AD+40+EX was significantly different from AD+40 (Bax, Bcl-2, cytochrome C, cleaved caspase-3, *P < .*001 each) and AD+EX exposure (Bax, Bcl-2, cytochrome c, cleaved caspase-3, *P <* .001 each), while AD+40 and AD+EX did not show an intergroup difference. When the TUNEL-positive cells of the hippocampus were compared, the AD group showed a significant increase compared to the CON group (*P <* .001). In contrast, when the AD group was compared with the treatment groups, a reduction was observed in the AD+40 (*P <* .001), AD+EX (*P <* .001), and AD+40+EX (*P <* .001) treated groups. When the treatment groups were compared among themselves, an intergroup difference was observed for AD+40+EX from AD+40 (*P =* .001) and AD+EX (*P =* .001) while AD+40 and AD+EX groups did not show an intergroup difference. Therefore, exercise under the exposure to 40-Hz light flickering was the most effective in decreasing apoptosis and cell death in the hippocampus, and this was restored to levels observed in the CON group (Fig. 5, Table 5).

**Table 5:** Effect of exercise under exposure to the 40-Hz light flickering on apoptosis and cell death in the hippocampus

Group	Bax	Bcl-2	Cytochrome c	Caspase-3	TUNEL (mm^2^)
CON	1.00 ± 0.14	1.00 ± 0.03	1.00 ± 0.05	1.00 ± 0.08	26.68 ± 2.63
AD	2.14 ± 0.11*	0.31 ± 0.05*	1.74 ± 0.07*	1.79 ± 0.08*	109.06 ± 5.92*
AD+40	1.60 ± 0.05*^#^	0.62 ± 0.05*^#^	1.42 ± 0.04*^#^	1.49 ± 0.04*^#^	59.59 ± 3.48*^#^
AD+EX	1.62 ± 0.07*^#^	0.68 ± 0.04*^#^	1.39 ± 0.04*^#^	1.41 ± 0.03*^#^	57.71 ± 4.34*^#^
AD+40+EX	0.99 ± 0.09*^#+^	1.29 ± 0.09*^#+^	0.90 ± 0.06*^#+^	0.96 ± 0.02*^#+^	32.99 ± 3.35*^#+^

*P < .05 compared to the CON group, ^#^*P* < .05 compared to the AD group, ^+^*P* < .05 compared to the AD+40 and AD+EX

### Effects of exercise under exposure to the 40-Hz light flickering on BDNF, PSD95, and synaptophysin in the hippocampus

**Fig. 6:**
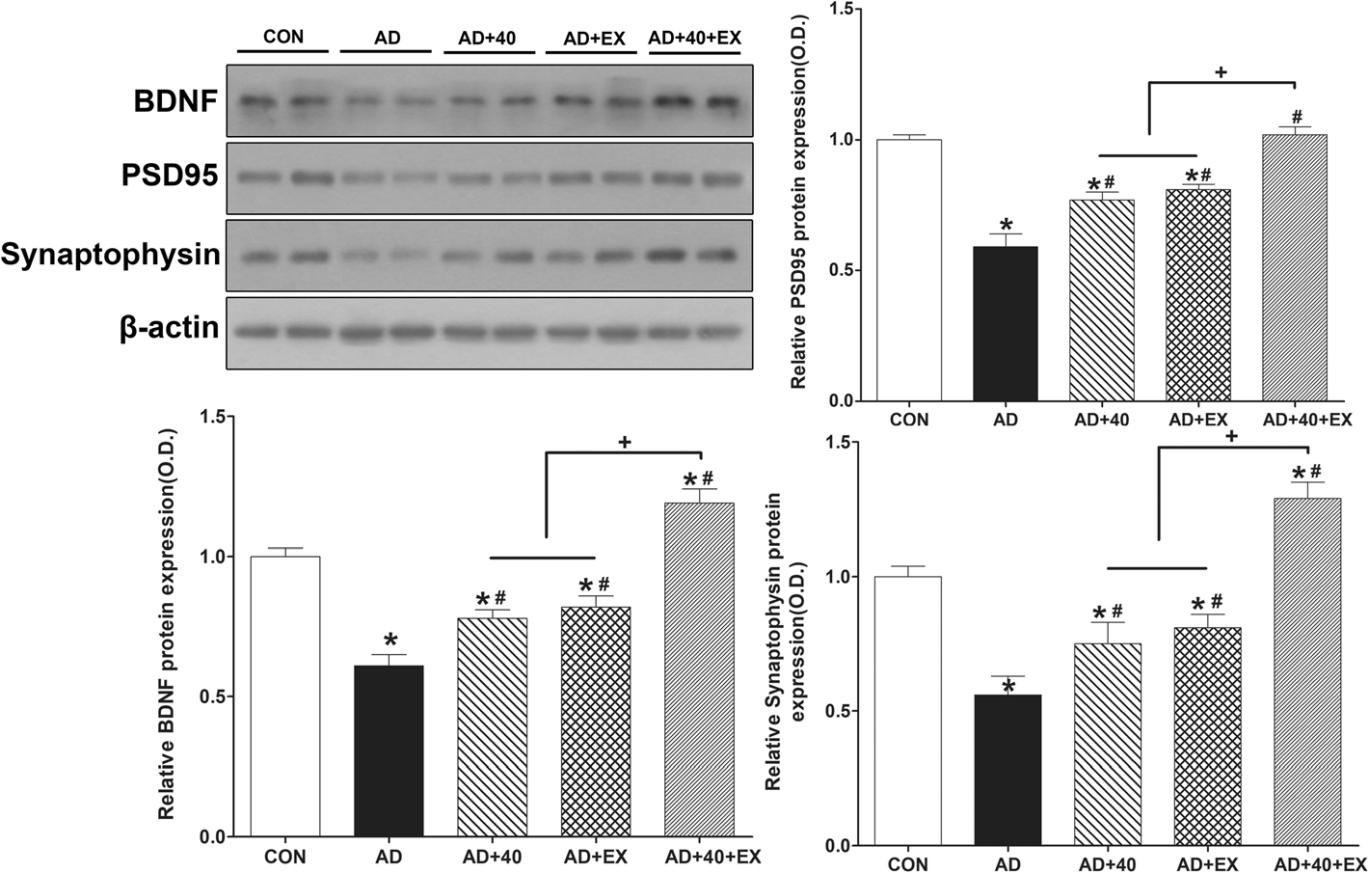
Effects of exercise under exposure to the 40-Hz light flicker on BDNF, PSD95, and synaptophysin in hippocampus. CON: wild-type, AD: 3xTg-AD, AD+40: 3xTg-AD and 40-Hz light flickering, AD+EX: 3xTg-AD and exercise, and AD+40+EX: 3xTg-AD and exercise in the 40-Hz light flickering. Data are expressed as the mean ± standard error of the mean (SEM). *P < .05 compared to the CON group. ^#^*P < .*05 compared to the AD group. ^+^*P < .*05 compared to the AD+40+EX

To examine changes in synaptic proteins in the hippocampus, the expression of BDNF, PSD 95, and synaptophysin were examined. For intergroup comparison, the value of the CON group was set to 1, which was compared to the relative value of each group. When the CON group was compared with the AD group, the expression of BDNF, PSD 95, and synaptophysin was reduced (*P <* .001 respectively). In contrast, when the AD group was compared with the treatment groups, AD+40 (BDNF: *P =* .024, PSD95: *P = .*002, synaptophysin: *P <* .001), AD+EX (BDNF: *P =* .005, PSD95: *P =* .001; synaptophysin: *P <* .001), and AD+40+EX (*P < .*001 respectively) groups showed a significant increase. In the comparison among treatment groups, AD+40+EX showed a significant difference in AD+40 and AD+EX (*P <* .001, respectively), while AD+40 and AD+EX treatments did not show any significant differences. Therefore, exercise under the exposure to 40-Hz light flickering was the most effective in improving the level of proteins associated with hippocampal synapses. In particular, the level of expression of BDNF (*P =* .007) and synaptophysin (*P <* .001) was higher than that of the CON group (Fig. 6, Table 6).

**Table 6:** Effect of exercise under exposure to the 40-Hz light flickering on BDNF, PSD95, and synaptophysin in the hippocampus

Group	BDNF	PSD95	Synaptophysin
CON	1.00 ± 0.03	1.00 ± 0.02	1.00 ± 0.04
AD	0.61 ± 0.04*	0.59 ± 0.05*	0.56 ± 0.07*
AD+40	0.78 ± 0.03*^#^	0.77 ± 0.03*^#^	0.75 ± 0.08*^#^
AD+EX	0.82 ± 0.04*^#^	0.81 ± 0.02*^#^	0.81 ± 0.05*^#^
AD+40+EX	1.19 ± 0.05*^#+^	1.02 ± 0.03*^#+^	1.29 ± 0.06*^#+^

*P < .05 compared to the CON group, ^#^*P* < .05 compared to the AD group, ^+^*P* < .05 compared to the AD+40 and AD+EX

### Effects of exercise under exposure to the 40-Hz light flickering on cell differentiation and neurogenesis in the hippocampus

**Fig. 7:**
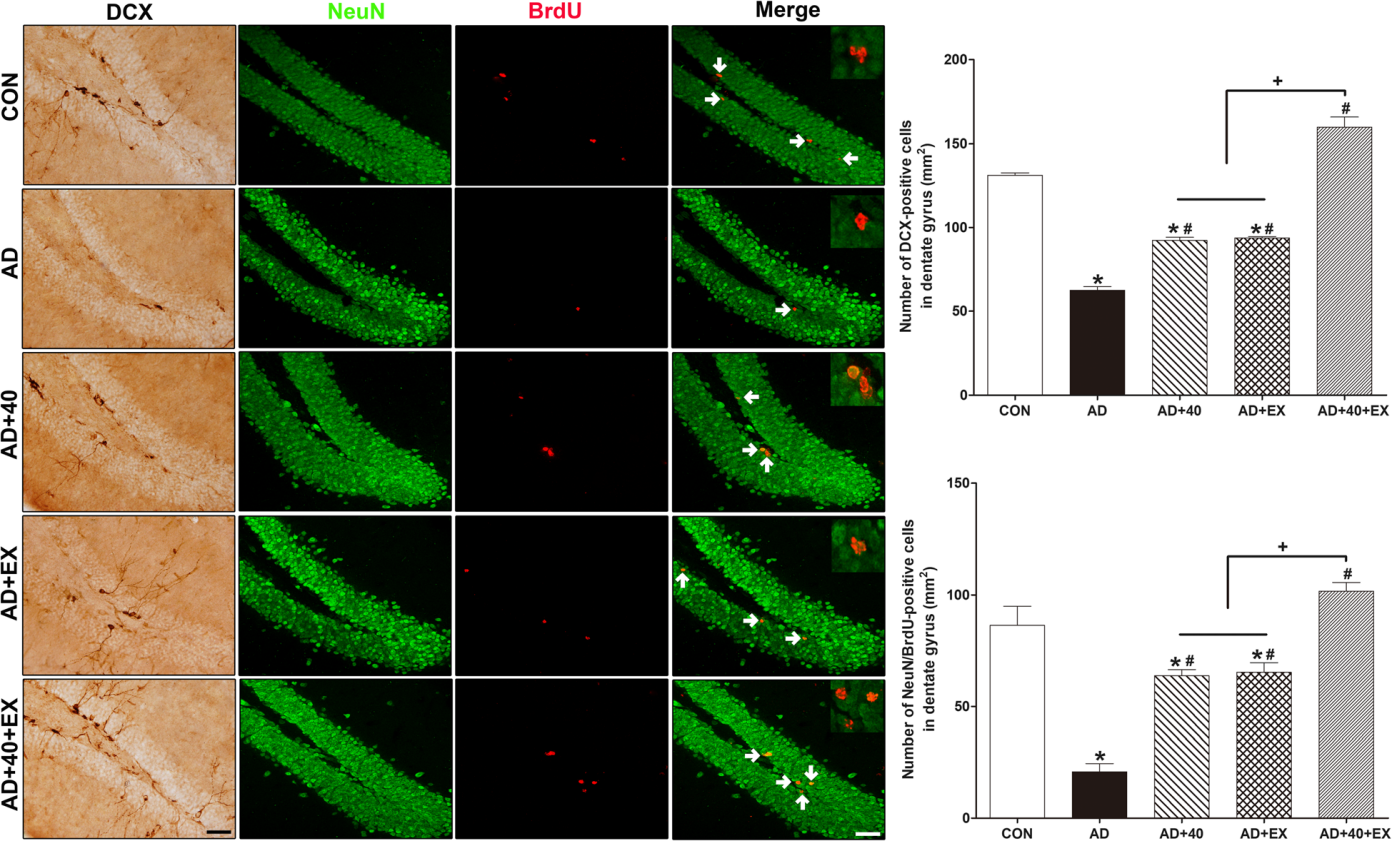
Effects of exercise under exposure to the 40-Hz light flicker on cell differentiation and neurogenesis in hippocampal dentate gyrus. The data are shown as the photomicrographs and data of DCX- and BrdU/NeuN-positive cells. The scale bar represents 50 μm. Mean ± standard error of the mean (SEM). CON: wild-type, AD: 3xTg-AD, AD+40: 3xTg-AD and 40-Hz light flickering, AD+EX: 3xTg-AD and exercise, and AD+40+EX: 3xTg-AD and exercise in the 40-Hz light flickering. Data are expressed as the mean ± standard error of the mean (SEM). *P < .05 compared to the CON group. ^#^*P < .*05 compared to the AD group. ^+^*P < .*05 compared to the AD+40+EX

To examine cell differentiation and neurogenesis in the hippocampus, DCX-positive cells and NeuN/brdU-positive cells were examined. Compared with the CON group, DCX-positive cells and NeuN/brdU-positive cells (*P < .*001 respectively) in the hippocampus decreased in the AD group. In contrast, a significant increase was observed for the AD+40 (DCX-positive cells: *P = .*006, NeuN/brdU-positive cells: *P = .*002), AD+EX (DCX-positive cells: *P = .*001, NeuN/brdU-positive cells: *P = .*002), and AD+40+EX (*P < .*001 respectively) groups. In the comparison of the treatment groups, AD+40+EX treatment showed a significant difference compared to AD+40 and AD+EX (*P < .*001, respectively), while AD+40 and AD+EX treatments did not show any significant difference. It is interesting to note that the level in AD+40+EX was increased and exceed that of the CON group (DCX-positive cells: *P = .*004; NeuN/brdU-positive cells: *P = .*003). Therefore, exercise under exposure to 40-Hz light flickering was the most effective in improving cell differentiation and neurogenesis in the AD hippocampus (Fig. 7, Table 7).

**Table 7:** Effect of exercise under exposure to the 40-Hz light flickering on cell differentiation and neurogenesis in the hippocampus

Group	DCX (mm^2^)	BrdU/NeuN (mm^2^)
CON	131.25 ± 1.38	83.38 ± 8.54
AD	62.50 ± 2.18*	20.79 ± 3.71*
AD+40	92.30 ± 1.90*^#^	63.75 ± 2.81*^#^
AD+EX	93.78 ± 0.80*^#^	65.30 ± 4.30*^#^
AD+40+EX	159.90 ± 6.07*^#+^	101.75 ± 3.88*^#+^

*P < .05 compared to the CON group, ^#^*P* < .05 compared to the AD group, ^+^*P* < .05 compared to the AD+40 and AD+EX

## Discussion

AD is an aging and neurodegenerative disorder characterized by deficits in learning, memory, and communication. AD accounts for 60 to 80% of dementia worldwide. The 3xTg AD animal model resembles the progression of cognitive and behavioral deficiencies seen in AD patients and is well suited for clinical studies of AD pathology. In this study, deficiencies in spatial learning, memory, and long-term memory were evaluated in the 3xTg AD group using the Morris Water maze and step through tests. In previous studies, 3xTg AD mice have shown loss of cognitive function and dementia-like behavioral and psychological symptoms along with in vivo long-term potentiation disorders, which are more severe in the older population [33]. This is particularly evident in the vulnerable areas of the brain, and the hippocampus is one of the most rapidly affected areas [34]. Signals that create our thoughts and memories are transmitted through nerve cells and the synapses between them. Aβ, an important protein for nerve cell growth and repair, is usually produced under physiological conditions and is excreted through the urine but when brain damage occurs, it is produced excessively and adheres to nerve cells and forms aggregates, blocking synapses, impeding the transmission of nerve signals, and causing inflammation and damage in neurons. When this occurs, memory dysfunction is a major symptom as the hippocampus and the temporal lobe are the first to develop abnormalities, which gradually spread to other areas of the brain. Altered cognitive function, including learning and memory deficits, are closely related to Aβ accumulation in the hippocampus. Aβ aggregation can cause synaptic dysfunction and neurodegeneration, which can impair cognitive function including spatial memory [35, 36]. In addition, Aβ stimulates glycogen kinase 3 (GSK3) to induce tau protein phosphorylation [37], and tau overexpression also causes an increased Aβ plaque accumulation [38]. GSK3, which promotes tau overexpression, is inhibited by phosphorylation of Ser21 by GSKα or Ser9 by GSK3β [39] and is increased in the absence of Akt activity [40]. Previous studies have shown that p-GSK3β (ser9) and p-Akt (ser473) were decreased while the expression of p-tau and Aβ increased simultaneously in the 3xTg-AD mouse [41, 42]. Aβ is closely associated with mitochondrial dysfunction, as evidenced by the accumulation of mitochondrial damage in the brains of AD patients [43], which may induce neuronal apoptosis [44], impair the movement of mitochondria, and cause synaptic degeneration by reducing mitochondrial length [45]. In the present study, the 3xTg-AD group with high Aβ expression also showed decreased mitochondrial function in the hippocampus such as decreased Ca^2+^ retention in hippocampal mitochondria and increased H_2_O_2_ production, a marker of reactive oxygen species (ROS). This deterioration led to an increase in cell death due to an increase in apoptosis and the expression of pro-apoptotic factors including Bax, cytochrome c, and caspase-3, as well as a decrease in the anti-apoptotic factor, Bcl-2. Cellular proliferation, neurogenesis, and synaptic markers BDNF, PSD95, and synaptophysin were also reduced in the hippocampus. Therefore, overexpression of Aβ and tau may decrease mitochondrial function, increase cell death, decrease neuron production, and reduce neuronal plasticity in the hippocampus. In previous studies, an increase in Aβ concentration resulted in increased secretion of H_2_O_2_, dysregulation of the cytosolic and mitochondrial Ca^2+^ homeostasis, and cytochrome c in the mitochondria [46, 47]. Particularly, mitochondrial Ca^2+^ overloading results in an increase of ROS production in mitochondria [48], the oxidative stress leads to abnormalities in the cell calcium storage, and the ability to control oxidative stress and respond to metabolic disorder links the AD-causing gene mutations to the disease process [49], while cell death was increased as were Bax levels with a concomitant decrease in Bcl-2 in the hippocampus of the 3xTg AD mouse, showing deficiencies in cognitive functions due to a decrease in BDNF, PSD95, and synaptophysin expression [50–52]. Aβ is an important pathological factor in AD. It is a neurotoxin, which can pathologically affect various brain cells. It seems that the decrease in cognitive function occurs due to inhibition of neuroplasticity following an alteration in mitochondrial function caused by excessive accumulation of Aβ and overexpression of tau in the hippocampus.

A new treatment method using LED has been recently suggested as a non-invasive therapy to treat AD. In particular, 40-Hz light flicker (LED flickers 40 times per second) is effective in stimulating the brain and restoring gamma rhythms. In an animal AD model, 40-Hz light flicker reduced p-tau and Aβ, which resolved memory abnormalities [53, 54]. Long-term visual stimulation using 40-Hz light flickering entrained gamma oscillations in the visual cortex, CA1 of the hippocampus, and the prefrontal cortex in Tau P301S and CK-p25 mice; as a result, spatial learning and memory and protein levels of various synaptic signaling and synaptic plasticity markers were improved [55]. Duan et al. [56] reported that the light from the LED inhibited apoptosis, which induces Aβ. The visual stimulation of 40-Hz light flickering has been shown to improve cognitive function by reducing neuronal and synaptic loss as well as amyloid plaques and tau phosphorylation in various AD mouse models (5XFAD, APP/PS1, P301S, and CK-p25) [50–52, 57]. In the present study, gamma oscillation was not measured, but in previous studies, it has been shown that the gamma oscillation in the hippocampus was altered with respect to time and concentration of Aβ [23], while the 3xTg-AD model showed synchronization of abnormal beta and gamma frequencies [58]. In the present study, the 40-Hz light flickering treatment group showed a reduction in tau phosphorylation and Aβ in the hippocampus and an improvement in spatial learning, memory, long-term memory, mitochondrial function, and neuroplasticity. This may suggest that the expression of tau and Aβ in the hippocampus was reduced by rescued gamma oscillations, which then led to an improvement in cognitive function. This may be due to the enhancement of mitochondrial function such as Ca^2+^ retention and ROS stabilization in the hippocampus, increased neuroplasticity such as the increase in proteins related to neurogenesis and synapses, and the inhibition of apoptosis.

Another non-invasive method to treat AD is exercise. Physical activity is known to promote brain health and improve cognitive functioning in the elderly; it has also been shown to increase hippocampal size and increase BDNF levels and neurogenesis [59, 60]. Exercise plays an important role in protecting against cognitive disorders due to dementia [61], and studies have reported low plasma Aβ and brain amyloid levels in people with low levels of physical activity [62]. In addition, in various AD animal models, exercise has been shown to delay or protect against the progression of AD by reducing Aβ and hyperphosphorylated tau protein [63–66], activating p-Akt and p-GSK3β, and reducing hyperphosphorylated tau levels [67]. As shown, exercise can play an important role in AD treatment including regulation of tau and Aβ in many of the previous studies. In addition, exercise-induced neuronal activity in the hippocampus requires increased mitochondrial capacity to produce ATP from oxidative phosphorylation of glucose. As a result, ROS may accumulate, but exercise may activate mitochondrial function and mitigate ROS-induced neurotoxins, and the protective effect of exercise against ROS production may be important in the hippocampus of patients with AD [68]. Exercise also improves hippocampal function by alleviating ROS such as H_2_O_2_ and inducing Ca^2+^ retention in hippocampal mitochondria under various neurotoxic conditions, and that mitochondrial function activated by exercise decreases apoptosis [69]. Furthermore, Aβ-dependent cell death is significantly suppressed after exercise in the hippocampus of an AD model [70]. In addition, exercise increases synaptic plasticity in the hippocampus, and Revilla et al. [71] found increased synaptophysin and PSD95 protein expression in the hippocampus of the 3xTg AD model.

In the present study, mitochondrial function and neuroplasticity were improved by Aβ and tau overexpression in an AD animal model through exercise as in the previous study. However, the therapeutic benefit of this in patients with AD remains controversial. Our study showed that exercise along with a non-invasive approach such as 40-Hz light flickering led to a significant improvement in AD patients, which was an important conclusion. Although much research on 40-Hz light flickering is still needed, Aβ and tau protein levels were suppressed, and the improvement in Aβ and tau expression caused by 40-Hz light flickering may have induced various positive cellular effects. Thus, under these circumstances, exercise may have a positive effect on this AD animal model as a complimentary therapy to 40-Hz light flickering.

## Conclusion

Many previous studies have suggested that Aβ and tau are important pathological factors in AD pathogenesis. Abnormal expression of Aβ and tau in the hippocampus may induce a variety of changes such as decreased mitochondrial function, apoptosis, decreased neurogenesis, and reduced synaptic-related proteins, which may cause a decline in cognitive functioning. However, if gamma oscillation can be stabilized through the visual stimulation of 40-Hz light flickering, exercise may improve cognitive functioning, as these two non-invasive methods produced a synergistic effect, which improved mitochondrial function and neuroplasticity by reducing Aβ and tau levels. Further research is needed to determine the effectiveness of these non-invasive methods in various models and their clinical applications.

## Data Availability

All data analyzed or generated during current study are included in this published article and available from the corresponding author on reasonable request.

## Abbreviations

3xTg-AD: Triple transgenic mouse model of Alzheimer’s disease
AD: Alzheimer’s disease
Aβ: Amyloid beta
BDNF: Brain-derived neurotrophic factor
BrdU: 5-Bromo-2′-deoxyuridine
DG: Dentate gyrus
GSK3β: Glycogen synthase 3 beta
LED: Light-emitting diode
PSD95: Postsynaptic density protein 95
ROS: Reactive oxygen species
TUNEL: Terminal deoxynucleotidyl transferase dUTP nick end labeling
